# Effects of the dietary protein-to-energy ratio on the growth performance, body composition, and health status of large-sized grass carp, *Ctenopharyngodon idella*

**DOI:** 10.3389/fphys.2025.1665511

**Published:** 2025-10-28

**Authors:** Zhangbin Liao, Lei Wang, Biao Yun, Xiaolin Dong, Fengchi Niu, Jian Wang, Xueqiao Qian, Xueming Hua

**Affiliations:** ^1^ Key Laboratory of Microecological Resources and Utilization in Breeding Industry, Ministry of Agriculture and Rural Affairs, Haid Central Research Institute, Guangdong Haid Group Co., Ltd., Guangzhou, Guangdong, China; ^2^ Centre for Research on Environmental Ecology and Fish Nutrition, Ministry of Agriculture and Rural Affairs, Shanghai Ocean University, Shanghai, China

**Keywords:** grass carp, *Ctenopharyngodon idella*, protein-to-energy ratio, growth performance, health status

## Abstract

A 56-day feeding trial was performed to investigate the effects of the dietary protein-to-energy (P/E) ratio on the growth performance, body composition, and health status of large-sized grass carp, *Ctenopharyngodon idella*. The fish (initial body weight 2,200.4 ± 79.3 g) were randomly fed one of the six isolipidic and isoenergetic diets (gross energy 10 kJ/g), which were formulated with various P/E ratios (21.7 mg/kJ, 23.7 mg/kJ, 24.9 mg/kJ, 27.1 mg/kJ, 29.2 mg/kJ, and 31.5 mg/kJ) and named P/E 21.7, P/E 23.7, P/E 24.9, P/E 27.1, P/E 29.2 mg/kJ and P/E 31.5, respectively. After the feeding trial, the best growth performance was observed in the P/E 29.2 group, which had the highest weight gain. In addition, fish fed the optimal P/E diet exhibited a superior health status in terms of tissue histology and biochemical analyses of serum and liver. The liver transcriptome assay revealed that a suitable P/E ratio potentially enhances growth performance and immune function by modulating the AMPK signaling pathway, the Ras signaling pathway, and arachidonic acid metabolism, along with affecting rRNA synthesis by regulating ribosome biogenesis gene expression in eukaryotes. Based on the second-order polynomial regression analysis of the growth performance and health status against P/E, the optimal P/E range was found to be 27.36–28.93.

## 1 Introduction

The rapid development of aquaculture has been driven by the increasing global demand for aquatic products. Aquafeed accounts for 50%–70% of aquaculture operational costs, largely due to the incorporation of a high percentage of protein needed for tissue growth, maintenance, and reproduction (Zehra and Khan, 2012; Gonçalves et al., 2018). From an economic standpoint, optimizing protein utilization for tissue synthesis rather than energy metabolism is critical. Carbohydrates can be used as the most economical source of energy for aquaculture animals. Adequate levels of carbohydrates, such as starch, can promote a protein-sparing effect, which ultimately results in an optimal cost/benefit ratio and a reduction in ammonia excretion (Enes et al., 2009; Pérez-Jiménez et al., 2015; Zhao et al., 2024). Many studies have implied that carbohydrate requirements vary among different fish species. With high intestinal amylase activity and an efficient blood glucose regulation mechanism, herbivorous fish can make good use of carbohydrates in their diet, and carbohydrate content in some fish diets can be as high as 40% (Mohapatra et al., 2003; Tian et al., 2012; Kamalam et al., 2017).

The grass carp, *Ctenopharyngodon idella*, is a typical herbivorous, agastric finfish and one of the most important species cultured in China (Jin et al., 2015). Current research predominantly focuses on individual macronutrients, such as protein and carbohydrates. Studies on protein-to-energy (P/E) ratio requirements have been limited primarily to young grass carp (Yu et al., 2022). Notably, there is a lack of studies addressing large-sized grass carp. To address this gap, it is imperative to conduct systematic investigations into the P/E ratio requirements specifically for large-sized grass carp.

The present study aimed to (1) evaluate the effects of the dietary P/E ratio on growth performance in large-sized grass carp; (2) analyze the biochemical parameters of liver, serum, and intestinal histology to evaluate the effects of the dietary P/E ratio on health status;, and (3) analyze the liver transcriptome profiles to explore the effects of the dietary P/E ratio on liver metabolism. These findings provide new insights into the P/E ratio requirements for large-sized grass carp.

## 2 Materials and methods

### 2.1 Experimental diets and fish

The proximate composition of the experimental diets is presented in Table 1. Six isolipidic and isoenergetic diets (gross energy 10 kJ/g) were formulated with various P/E ratios (P/E 21.7 mg/kJ, P/E 23.7 mg/kJ, P/E 24.9 mg/kJ, P/E 27.1 mg/kJ, P/E 29.2 mg/kJ, and 31.5 mg/kJ) and named P/E 21.7, P/E 23.7, P/E 24.9, P/E 27.1, P/E 29.2, and P/E 31.5, respectively. All the ingredients were obtained from Guangdong Haid Group Co., Ltd. (China). The diets were prepared, packed, and stored following the procedures of a previous study (Dong et al., 2019).

**TABLE 1:** Formulation and composition of experimental diets (% on a dry matter basis).

Ingredients	P/E 21.7	P/E 23.7	P/E 24.9	P/E 27.1	P/E 29.2	P/E 31.5
Soybean meal	17.2	17.2	17.2	17.2	22.2	27.2
Rapeseed meal	12.5	18.5	24.5	30.5	30.5	30.5
Distillers dried grains with solubles	10.0	10.0	10.0	10.0	10.0	10.0
Wheat meal	48.0	42.0	36.0	30.0	25.0	20.0
Soybean oil	1.50	1.50	1.50	1.50	1.50	1.50
Soya lecithin	1.50	1.50	1.50	1.50	1.50	1.50
Monocalcium phosphate	3.00	3.00	3.00	3.00	3.00	3.00
Sodium chloride	0.10	0.10	0.10	0.10	0.10	0.10
Choline chloride	0.13	0.13	0.13	0.13	0.13	0.13
Vitamin premix^a^	0.50	0.50	0.50	0.50	0.50	0.50
Mineral premix^b^	1.00	1.00	1.00	1.00	1.00	1.00
Antioxidants	0.02	0.02	0.02	0.02	0.02	0.02
Calcium	0.05	0.05	0.05	0.05	0.05	0.05
Bentonite	4.50	4.50	4.50	4.50	4.50	4.50
Proximate composition						
Crude protein	22.8	24.4	25.4	27.0	28.9	30.6
Crude lipid	6.22	6.06	6.30	6.27	6.16	6.19
Energy (kJ/g)^c^	10.5	10.3	10.2	9.98	9.88	9.71
Protein/energy (P/E, (mg/kJ))	21.7	23.7	24.9	27.1	29.2	31.5

^a^ Vitamin premix (mg/kg diet): thiamin, 20; riboflavin, 20; pyridoxine, 20; cyanocobalamin, 0.02; folic acid, 5; calcium pantothenate, 50; inositol, 100; niacin, 100; biotin, 0.1; starch, 645.2; ascorbic acid, 100; vitamin A, 110; vitamin D, 20; vitamin E, 50; vitamin K, 10.

^b^ Mineral premix (mg/kg diet): NaCl, 500; MgSO_4_·7H_2_O, 4,575; NaH_2_PO_4_·2H_2_O, 12,500; KH_2_PO_4_, 16,000; Ca(H_2_PO_4_)_2_·H_2_O, 6,850; FeSO_4_, 1,250; C_6_H_10_CaO_6_·5H_2_O, 1750; ZnSO_4_·7H_2_O, 111; MnSO_4_·4H_2_O, 61.4; CuSO_4_·5H_2_O, 15.5; CoSO_4_·6H_2_O, 19.02; KI, 178.33; corn starch, 6,253.33.

^c^ Estimated energy was calculated based on 16.9 kJ/g protein, 37.6 kJ/g lipid, and 16.7 kJ/g carbohydrate.

This feeding trial was conducted at the Guangdong Haid Group Co., Ltd., Seagull Island aquaculture base. Prior to the experiment, the experimental fish were acclimated in a large net cage (8 m × 16 m × 2 m) in the pond and fed a commercial feed for 30 days. Following a 24-h fasting period, fish were randomly distributed into 24 net cages (4 m × 4 m × 2 m). Each cage contained 10 fish (initial body weight approximately 2,200 g). Each experimental feed was randomly assigned to one of the four cages. The fish were reared for 8 weeks and fed three times daily at 7:00, 12:00, and 17:00. Water quality parameters were maintained within the following ranges: temperature, 27–32 °C; ammonia nitrogen, 0.2–0.6 mg/L; dissolved oxygen, 5–7 mg/L; pH, 6.7–7.0.

At the end of the feeding experiment, the fish were fasted for 24 h. After that, they were exposed to MS222 (Sigma-Aldrich, St. Louis, MO, United States) with a concentration of 0.2% (w/v) for 5 min until cessation of opercular movement. The number and weight of fish per cage were recorded. Two fish were randomly selected from each cage for serum, liver, intestinal, and muscle sampling. The remaining fish were returned to the cage to recover. Blood was collected from the tail vein and allowed to clot at room temperature for 2 h, then placed at 4 °C for 6 h. After centrifugation (836 g, 10 min, 4 °C), the supernatant was collected as serum samples. After dissection, before collecting tissue samples, the weight and body length of two randomly selected fish were recorded, along with the weights of the liver and viscera, to calculate the hepatosomatic index (HSI), viscerosomatic index (VSI), and condition factor (CF). Two small liver tissue samples (from the liver tip) and two muscle tissue samples (from the dorsal muscle, measuring approximately 3 cm × 1.5 cm) were quickly frozen in liquid nitrogen and then stored at −86 °C. Additionally, two small liver tissue samples (from the liver tip) and two midgut tissue samples were immediately fixed in 4% formaldehyde, followed by standard tissue processing for dehydration, paraffin embedding, sectioning, and HE staining (hematoxylin–eosin) for microscopic observation and photography of liver and intestinal tissue morphology.

All experimental procedures were performed in strict accordance with the Management Rule of Laboratory Animals (Chinese Order No. 676 of the State Council, revised 1 March 2017).

### 2.2 Proximate composition of diets and tissues and biochemical parameters of liver and serum

The proximate composition of diets and tissues was analyzed with the Association of Official Analytical Collaboration (AOAC) standard methods. For the analysis of moisture, samples were dried in a 105 °C oven until the weight was constant. Ash, crude protein, and crude lipid content were assayed by 550 °C incineration (8 h), the Kjeldahl method (FOSS 2300), and the chloroform–methanol method, respectively.

Serum and liver biochemical indices, such as alanine aminotransferase (ALT), superoxide dismutase (SOD), total antioxidant capacity (T-AOC), malondialdehyde (MDA), triacylglycerol (TG), total cholesterol (T-CHO), high-density lipoprotein cholesterol (HDL-C), low-density lipoprotein cholesterol (LDL-C), hepatic glycogen, and serum glucose, were analyzed using commercial kits (Nanjing Jiancheng Bioengineering Institute, Nanjing, China).

α-amylase, trypsin, and lipase in the intestine were analyzed using commercial kits (Beijing Solarbio Science and Technology Co., Ltd., Beijing, China).

### 2.3 RNA isolation, cDNA library construction, and Illumina sequencing

The detailed methods for RNA isolation, cDNA library construction, and sequencing were described previously (Liao et al., 2024). Pooled samples from two individual fish from each cage were used (groups P/E 21.7 and P/E 29.2 were used as characteristic groups). The data processing and enrichment analysis of differentially expressed genes (DEGs) between groups followed the method used by Liao et al. (2024).

### 2.4 Calculations and statistical analysis

$$\begin{array}{l} \text{Weight gain rate }\left(\text{WGR},\%\right)=\left(\text{final body weight}-\text{initial body weight}\right)\;/\\ \;\text{ initial body weight}\times 100 \end{array}$$

$$\text{Feed conversion ratio }\left(\text{FCR}\right)=\text{feed consumption }/\text{ body weight gain}$$

$$\begin{array}{ll} \text{Condition factor }\left(\text{CF},g/{\text{cm}}^{3}\right) & =\left(\text{final body weight }/\text{ final body }{\text{length}}^{3}\right)\\ & \;\times 100 \end{array}$$

$$\text{Hepatosomatic index }\left(\text{HSI},\%\right)=\left(\text{liver weight }/\text{ body weight}\right)\times 100$$

$$\begin{array}{ll} \text{Viscerosomatic index }\;\left(\text{VSI},\%\right) & =\left(\text{viscera weight }/\text{ body weight}\right)\\ & \;\times 100 \end{array}$$

$$\begin{array}{ll} \text{Liposomatic index }\left(\text{LSI},\%\right) & =\left(\text{abdominal lipid weight }/\text{ body weight}\right)\\ & \;\times 100 \end{array}$$

All statistical analyses were conducted using SPSS 25.0 (IBM, United States). All data are reported as the mean ± standard error of the mean (SEM). All data were analyzed using one-way analysis of variance (ANOVA), followed by a Tukey’s multiple range test or an independent sample t-test. Differences were considered statistically significant when *P* < 0.05.

## 3 Results

### 3.1 Growth performance, somatic indices, and body composition

**FIGURE 1:**
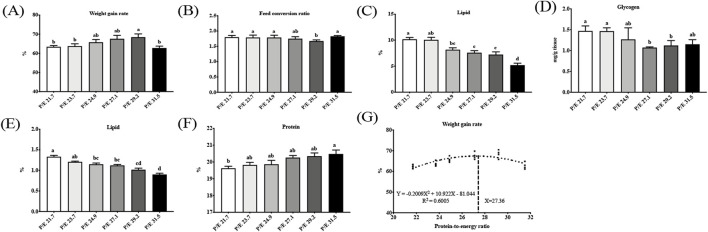
Effects of dietary protein-to-energy (P/E) ratios on the growth performance and tissue composition of large-sized grass carp [**(A)** weight gain rate; **(B)** feed efficiency ratio; **(C)** and **(D)** liver lipid and glycogen content (of wet weight, ww); **(E)** and **(F)** muscle lipid and protein content (ww), and **(G)** relationship of weight gain rate with dietary P/E, respectively] of large-sized grass carp. Data bars with different superscripts are significantly different (P < 0.05).

In the present study, no fish mortality was found in any group. As the P/E ratio increased, the weight gain rate increased up to P/E 29.2, after which it decreased; the weight gain rate of the P/E 29.2 group was significantly higher than that of the P/E 21.7, P/E 23.7, and P/E 31.5 groups (*P* < 0.05) (Figure 1), while the feed conversion ratio showed an opposite trend. No significant differences (*P* > 0.05) were observed in HSI, VSI, LSI, or CF (Supplementary Figure S1). Based on the second-order polynomial regression analysis of the weight gain rate against P/E, the optimal P/E level was 27.36 (Figure 1).

As the P/E ratio increased, the liver lipid content showed a downward trend, and the glycogen content decreased and then stabilized, while there were no significant differences (*P* > 0.05) in liver moisture and protein content. Muscle lipid content showed a downward trend with increasing P/E ratios, while protein content showed the opposite trend. There was no significant difference (*P* > 0.05) in moisture content.

### 3.2 Biochemical parameters

**FIGURE 2:**
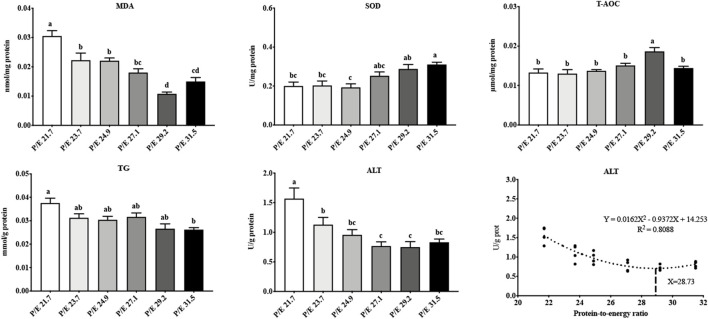
Effects of dietary P/E ratios on the liver biochemical parameters of large-sized grass carp. Data bars with different superscripts are significantly different (P < 0.05).

In the liver, as the P/E ratio increased, the content of MDA decreased down to P/E 29.2, after which it increased (Figure 2), while the SOD activity showed an overall upward trend. T-AOC was highest in the P/E 29.2 group and was significantly higher than that in the other groups (*P* < 0.05). ALT activity decreased with an increase in the dietary P/E ratio and then stabilized. The TG content showed a decreasing trend. No significant differences (*P* > 0.05) in T-CHO, HDL-C, LDL-C, or PC content were found among the groups (Supplementary Figure S2). Based on the second-order polynomial regression analysis of ALT activity against P/E, the optimal P/E level was 28.93 (Figure 2).

**FIGURE 3:**
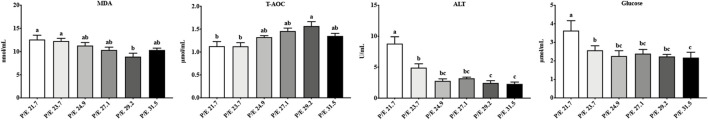
Effects of dietary P/E ratios on the serum biochemical parameters of large-sized grass carp. Data bars with different superscripts are significantly different (P < 0.05).

The MDA, T-AOC, and ALT activities in serum showed the same trend as in the liver, while glucose levels showed an overall decreasing trend with the increase of the dietary P/E ratio (Figure 3). No significant differences (*P* > 0.05) in T-CHO, HDL-C, TG, or PC content were found among the groups.

**FIGURE 4:**
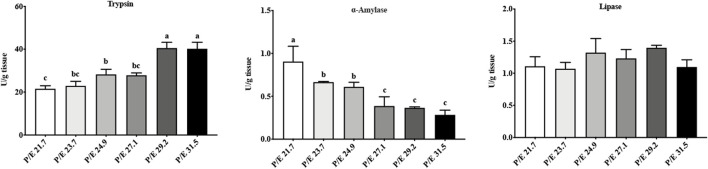
Effects of dietary P/E ratios on the intestinal enzyme activity of large-sized grass carp. Data bars with different superscripts are significantly different (P < 0.05).

In the intestine, trypsin activity increased with the increase in the dietary P/E ratio, reaching a maximum value in the P/E 29.2 group, and then, no further increase (Figure 4) was noted. As the dietary P/E ratio increased, α-amylase activity showed a continuous downward trend. No significant difference (*P* > 0.05) in lipase activity was noted among the groups.

### 3.3 Histological structure of tissues

**FIGURE 5:**
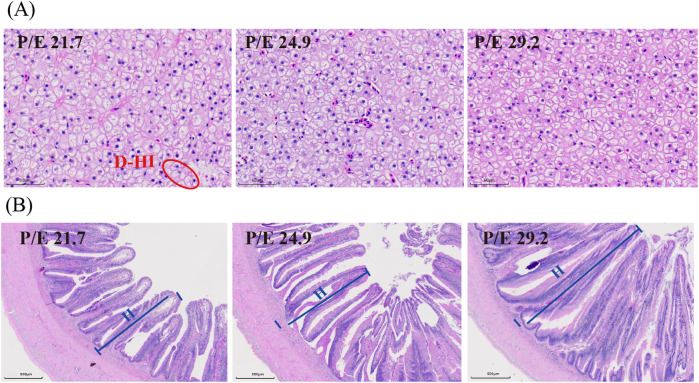
Effects of dietary P/E ratios on the tissue histology of large-sized grass carp. **(A)** and **(B)** Representative histology of liver and intestine, respectively. The red circles show necrosis of the liver parenchyma, namely, the damage to hepatocyte integrity (D-HI). The blue lines show the height (H) of the intestinal villi.

Liver histology indicated that liver cells became smaller and intracellular lipid content decreased with the increase in dietary P/E ratio (Figure 5A). Increased villus quantity and height were observed in the intestinal histology (Figure 5B).

### 3.4 Transcriptomic results

**FIGURE 6:**
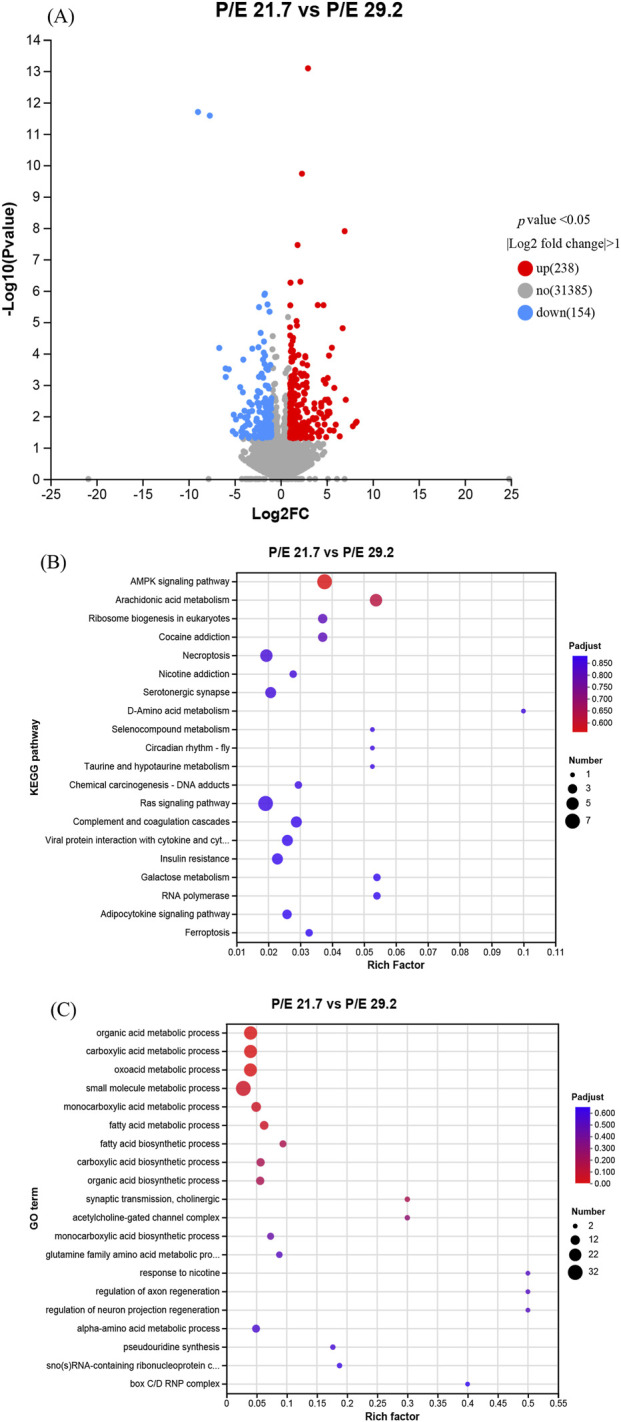
Effects of dietary P/E ratios on the liver transcriptome of large-sized grass carp. **(A)**, **(B)** and **(C)** Representative volcano plot of liver gene expression and KEGG and GO pathway enrichment for differentially expressed genes (DEGs) between the P/E 21.7 and P/E 29.2 groups, respectively. The spot size represents the number of DEGs significantly enriched in a pathway. P-adjust is the corrected P-value.

Liver samples from the P/E 21.7 and P/E 29.2 groups were used for transcriptomic analysis. A total of 392 genes were differentially expressed (*P* value <0.05; fold change >2) between the P/E 21.7 and P/E 29.2 groups. Compared to the P/E 21.7 group, the P/E 29.2 group up-regulated the transcription of 238 genes and down-regulated that of 154 genes (Figure 6A). The DEGs were mostly enriched in Kyoto Encyclopedia of Genes and Genomes (KEGG) pathways, such as the AMPK signaling pathway, arachidonic acid metabolism, Ras signaling pathway, ribosome biogenesis in eukaryotes, and necroptosis (Figure 6B), and in Gene Ontology (GO) terms such as organic acid metabolic process and small molecule metabolic process (Figure 6C).

## 4 Discussion

The protein-to-energy (P/E) ratio represents a more reasonable approach for defining protein requirements in fish than specifying crude protein levels alone (National Research Council, 2011; Liu et al., 2023). The National Research Council (National Research Council, 2011) recommends a suitable P/E ratio range of 19–27 mg/kJ for the majority of fish species. In the present study, the dietary P/E ratio was varied from 21.7 to 31.5 by increasing the protein content and decreasing the starch content. After an 8-week feeding trial, the WGR showed an overall upward trend and then decreased with increasing dietary protein levels. A similar phenomenon has been observed in the studies of the dotted gizzard shad *Konosirus punctatus* (Liu et al., 2023), the grass carp *Ctenopharyngodon idella* (Jin et al., 2015; Yu et al., 2022), the tilapia *Oreochromis niloticus* (Wu et al., 2021), the striped surubim *Pseudoplatystoma reticulatum* (Silva et al., 2019), the fingerling *Channa punctatus* (Zehra and Khan, 2012), and the obscure pufferfish *Takifugu obscurus* (Ye et al., 2017). The observed growth reduction at high P/E ratios may be attributed to impaired protein metabolism, an increased nitrogen metabolism burden, and energetic inefficiency (Kiron et al., 1995; McGoogan and Gatlin, 1999; Zehra and Khan, 2012). Conversely, the FCR showed an opposite trend to the WGR. Notably, hepatic lipid content decreased with an increasing P/E ratio, accompanied by reduced hepatocyte size and intracellular lipid deposition. An isoenergetic experimental design necessitated a reduction in dietary starch as protein content increased. In the present study, grass carp fed high-starch diets accumulated greater glycogen deposition in their livers. These results are in agreement with those of several previous studies on Nile tilapia (Gaye-Siessegger et al., 2006), grass carp (Jin et al., 2015), and hybrid grouper *Epinephelus fuscoguttatus* ♀ × *Epinephelus lanceolatus* ♂ (Jiang et al., 2016), which found that a high-protein/low-carbohydrate diets lead to significantly higher lipid gains and apparent lipid conversion values. Another study reported that a low-protein/high-carbohydrate diet could increase the amount of acetyl coenzyme A and dihydroxyacetone phosphate during glycolysis, and more acetyl coenzyme A and dihydroxyacetone phosphate were used for lipid synthesis in obscure pufferfish (Ye et al., 2017). Unexpectedly, no significant difference was observed in body condition indices was observed with decreasing liver lipid content, which was in accordance with a previous study (Jiang et al., 2016).

A histological structure change that followed the P/E ratio was observed in the present study. In the low P/E ratio groups, hepatocyte vacuolization was observed in H&E-stained livers, suggesting lipid accumulation and potential metabolic dysfunction. This finding aligns with that of Zhao et al. (2023), who reported that a low P/E ratio induces hepatic structural damage and subsequent inflammatory responses, likely due to excessive lipid deposition resulting from impaired energy metabolism. Similar results have been reported in other fish species (Camargo and Martinez, 2007; Sun et al., 2019; Taj et al., 2023) and supporting the hypothesis that a suboptimal P/E ratio disrupts hepatic homeostasis. In addition, intestinal villus height and density significantly increased in groups fed an optimal P/E ratio, indicating an expanded absorptive surface area and enhanced digestive efficiency. These morphological improvements are consistent with previous studies in fish, which attributed such changes to balanced nutrient utilization and improved gut health under nutritionally adequate conditions (Xu et al., 2016; Taj et al., 2023; Zhao et al., 2024).

High protein levels in a diet stimulate proteolytic secretion in some fish species (Péres et al., 1998; Krogdahl et al., 2003; Bakke et al., 2011). In addition, higher levels of enzyme activities are related to better growth performance and higher feed utilization in fish (Furné et al., 2005). In the present study, grass carp fed diets with a protein content that ranged from 22.8% to 30.6% showed a progressive increase in intestinal trypsin activity as protein content in the diet rose, reaching a plateau at 28.9% protein. Similar results were found in gilthead sea bream *Sparus aurata* (García-Meilán et al., 2013). With the increased dietary P/E ratio, α-amylase activity decreased. It is easy to understand how adjusting the protein and starch content of the isoenergetic feed used in this study works: a higher P/E ratio indicates more protein and less starch in the feed.

A variety of diets with different dietary P/E ratios were designed in the current study to determine the optimal diet for maintaining the health status of grass carp. SOD and T-AOC are important components of the antioxidant defense system in fish, whereas the main product of lipid peroxidation, MDA, is a key indicator of oxidative damage (Meng et al., 2017; Liu et al., 2021; Guo et al., 2023). Analysis of the livers and sera of the fish revealed that SOD and T-AOC activities, coupled with MDA levels, indicate that an appropriate dietary P/E ratio enhances the antioxidant capacity of grass carp. ALT, an enzyme predominantly localized in liver parenchymal cells, is a well-established clinical indicator of hepatic function and health. ALT levels in livers and sera indicated that a low-protein and high-starch diet was not suitable for grass carp. This finding is in agreement with earlier works (Jin et al., 2015; Wang et al., 2018).

To elucidate the metabolic response of grass carp to dietary P/E ratios, a hepatic transcriptome analysis was conducted comparing groups fed P/E 21.7 and P/E 29.2 diets. Pathway enrichment analysis (KEGG and GO) identified the AMPK signaling pathway as the most significantly enriched cluster among DEGs (Supplementary Table S1). The AMPK signaling pathway is known to play an important role in the regulation of energy metabolic pathways in fish (Wu et al., 2016). Several genes were up-regulated in the P/E 29.2 group, including fatty acid synthase (*fasn*), which is an important rate-limiting enzyme involved in the lipogenesis pathway (Zheng et al., 2013), stearoyl-CoA desaturase (*scd*), which catalyzes the insertion of a cis double bond at the delta-9 position into fatty acyl-CoA substrates, glucose-6-phosphatase catalytic subunit 1b (*g6pc1b*), which hydrolyzes glucose-6-phosphate to glucose in the endoplasmic reticulum, and Ras-related protein Rab-2A (*rab2a*), which modulates the liver lipid accumulation (Morohoshi et al., 2021; Chen et al., 2022). However, carnitine palmitoyltransferase 1Ab (*cpt1ab*, a marker gene of mitochondrial fatty acid β-oxidation) and 6-phosphofructo-2-kinase/fructose-2,6-bisphosphatase 1 (*pfkfb1*), which are involved in the synthesis and degradation of fructose 2,6-bisphosphate, were down-regulated in the P/E 29.2 group. These expression patterns indicate that the P/E 21.7 diet suppressed lipogenesis and enhanced lipolysis to mitigate lipid accumulation, while simultaneously promoting glycolysis-driven *de novo* lipogenesis, which elevated hepatic TG content. This paradoxical metabolic shift aligns with the observation of increased TG content in the liver. Similar energy metabolism results were observed in Amur sturgeon *Acipenser schrenckii* after feeding them a low-protein, high-starch diet (Zhang et al., 2023). In addition to lipid and carbohydrate metabolism, the DEGs were also enriched in other GO terms such as organic acid metabolic process, carboxylic acid metabolic process, oxoacid metabolic process, and monocarboxylic acid metabolic process.

The Ras signaling pathway was the second largest cluster of DEG enrichment in KEGG pathways. As a core regulatory mechanism governing proliferation, survival, growth, differentiation, and inflammation, this pathway exhibited significant upregulation of key genes in the P/E 29.2 group (Wennerberg et al., 2005; Lee et al., 2002; Shaker et al., 2023). The expression of Ras signaling pathway-related genes such as Ras-related protein Ral-A-like (*rala*), which is involved in a variety of cellular processes including gene expression, cell migration, and proliferation (Cascone et al., 2008; Balasubramanian et al., 2010; Hiatt et al., 2018), phospholipase C, epsilon 1 (*plce1*), which participates in multiple signaling pathways affecting cell survival, cell growth, actin organization, and T-cell activation (Bunney and Katan, 2006; Abdou et al., 2022), ral guanine nucleotide dissociation stimulator (*ralgds*, a guanine nucleotide exchange factor activating either RalA or RalB GTPases and playing a crucial role in intracellular transport), colony-stimulating factor 1 receptor, alpha (*csf1ra*), which plays an important role in innate immunity and inflammatory processes, and phospholipase A2 group 10 (*pla2g10*), which may be involved in maturation and activation of innate immune cells (Nolin et al., 2017), were up-regulated in the P/E 29.2 group. This coordinated upregulation suggests that an optimal dietary P/E ratio enhances growth performance and immune function. Furthermore, the up-regulated cytosolic phospholipase A_2_ gamma-like (*pla2g4c*) in the P/E 29.2 group, known to regulate endoplasmic reticulum homeostasis and lipid droplet formation (Hanasaki, 2002; Linkous and Yazlovitskaya, 2010; Su et al., 2017), indicates enhanced lipid homeostasis at an optimal dietary P/E ratio. Conversely, the down-regulation of angiopoietin-1-like (*angpt1*), which mediates endothelial–matrix interactions, is an unresolved aspect requiring further investigation (d'Apolito et al., 2019).

Arachidonic acid metabolism and ribosome biogenesis in eukaryotes were also regulated by the dietary P/E ratio. The expression of arachidonic acid metabolism genes, such as *pla2g10*, *pla2g4c*, cytochrome P450 2J4-like (*cyp2j4*), which catalyzes the hydroxylation of carbon-hydrogen bonds (Zhang et al., 1997), gamma-glutamyltransferase 1 alpha (*ggt1a*), which is involved in arachidonic acid metabolism (Gong et al., 2024), and hydroperoxide isomerase ALOXE3-like, which oxygenates polyunsaturated fatty acids, was up-regulated in the P/E 29.2 group. These results indicate that a suitable dietary P/E ratio can increase fatty acid metabolism and enhance immune performance. The expression of ribosome biogenesis genes such as the GAR1 homolog, ribonucleoprotein (*gar1*), which is responsible for 18S rRNA production and rRNA pseudouridylation (Spaulding et al., 2022), the SNU13 homolog, small nuclear ribonucleoprotein b (*snu13b*, a component of the spliceosome and rRNA processing machinery); and the NOP58 ribonucleoprotein homolog (*nop58*), which is crucial for rRNA processing and assembly, were up-regulated in the P/E 29.2 group. Generally, the vigorous synthesis of rRNA indicates the demand for efficient and large-scale protein synthesis during cell proliferation. In the present study, the up-regulated genes involved in ribosome biogenesis in eukaryotes may partly explain the increased growth performance in the group P/E 29.2.

## 5 Conclusion

The present study indicated that a dietary protein-to-energy (P/E) ratio of 29.2 was optimal for large-sized grass carp, as it maximized growth performance and health status, as evidenced by superior weight gain, favorable hematological parameters, and improved tissue histology and hepatic transcriptome profiles. At the transcriptional level, the optimal P/E ratio enhanced growth performance and immune function by interrupting the AMPK signaling pathway, the Ras signaling pathway, and arachidonic acid metabolism, in addition to affecting rRNA synthesis via regulating the ribosome biogenesis gene expression in eukaryotes. Based on the second-order polynomial regression analysis of the growth performance and health status against the P/E ratio, the optimal P/E range was found to be 27.36–28.93. These findings provide novel insights into the nutritional requirements of large-sized grass carp, offering valuable guidance for optimizing feed formulations to enhance aquaculture productivity.

## Data Availability

The datasets presented in this study can be found in online repositories. The names of the repository/repositories and accession number(s) can be found in the article/Supplementary Material.
