# Supplementary figures and images for: Effects of the dietary protein-to-energy ratio on the growth performance, body composition, and health status of large-sized grass carp, Ctenopharyngodon idella
Source: Front Physiol. 2025 Oct 28;16:1665511. doi: 10.3389/fphys.2025.1665511 (PMC12602221; doi:10.3389/fphys.2025.1665511)

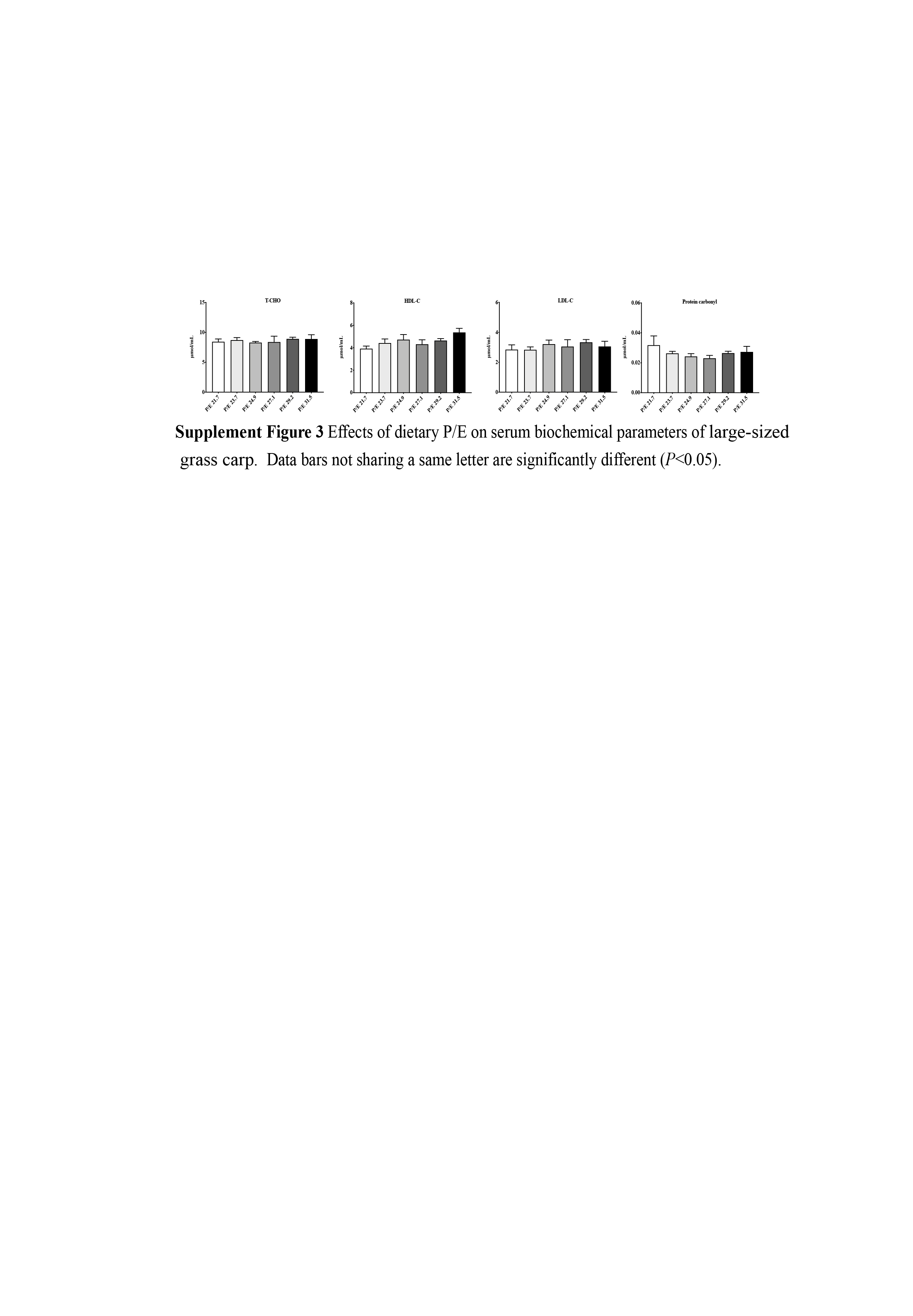

Supplement: Supplementary file 1 [file Image3.jpg]

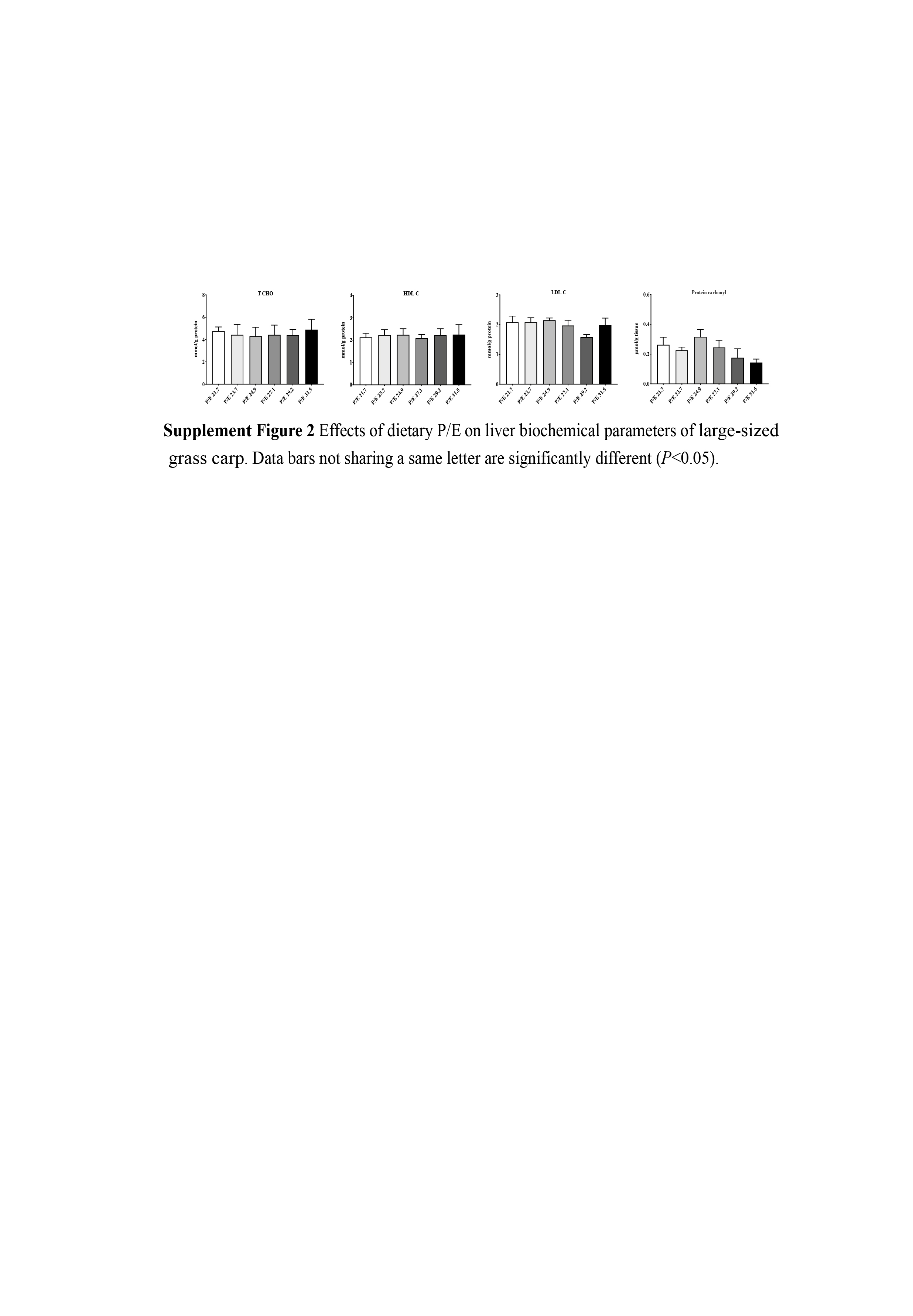

Supplement: Supplementary file 2 [file Image2.jpg]

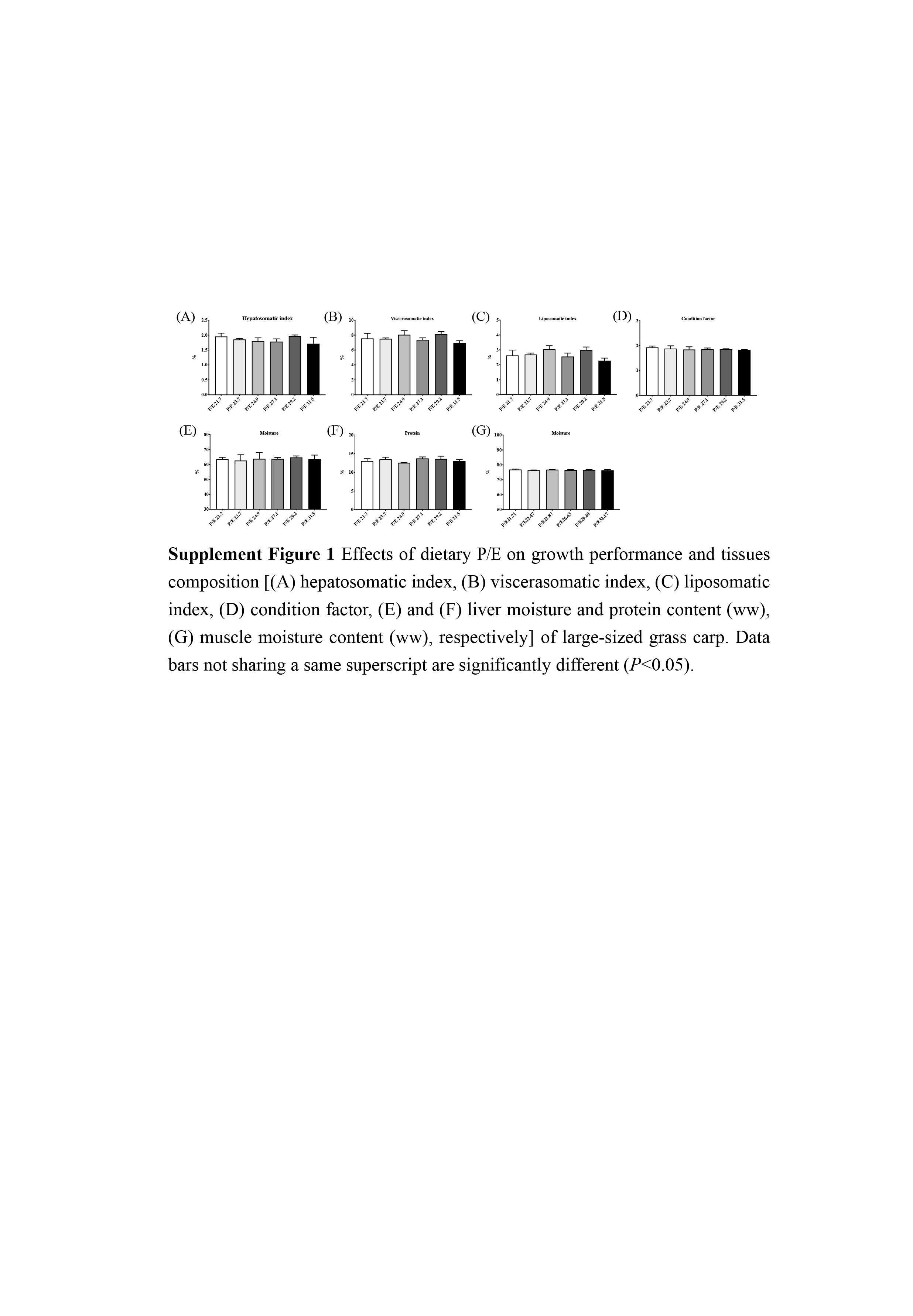

Supplement: Supplementary file 4 [file Image1.jpg]
