# Supplementary material for: Effects of the dietary protein-to-energy ratio on the growth performance, body composition, and health status of large-sized grass carp, Ctenopharyngodon idella
Source: Front Physiol. 2025 Oct 28;16:1665511. doi: 10.3389/fphys.2025.1665511 (PMC12602221; doi:10.3389/fphys.2025.1665511)
Supplement: Supplementary file 3 [file Table1.docx]

**Supplementary Table S1** The primary DEGs between group P/E 21.7 and P/E 29.2, which were mentioned in the discussion section.

| **Metabolic process**  **(GO term or KEGG pathway)** | **Gene abbreviation** | **Gene name** | **Gene function** |
| --- | --- | --- | --- |
| AMPK signaling pathway | *fasn* | fatty acid synthase ↑ | an important rate-limiting enzyme involved in lipogenesis pathway |
|  | *scd* | stearoyl-CoA desaturase (delta-9-desaturase) ↑ | catalyzes the insertion of a cis double bond at the delta-9 position into fatty acyl-CoA substrates |
|  | *g6pc1b* | glucose-6-phosphatase catalytic subunit 1b ↑ | hydrolyzes glucose-6-phosphate to glucose in the endoplasmic reticulum |
|  | *rab2a* | ras-related protein Rab-2A ↑ | modulates the liver lipid accumulation |
|  | *cpt1ab* | carnitine palmitoyltransferase 1Ab ↓ | a marker gene of mitochondrial fatty acid β-oxidation |
|  | *pfkfb1* | 6-phosphofructo-2-kinase/fructose-2,6-biphosphatase 1 ↓ | synthesis and degradation of fructose 2,6-bisphosphate |
| Ras signaling pathway | *rala* | ras-related protein Ral-A-like ↑ | involved in a variety of cellular processes including gene expression, cell migration, cell proliferation |
|  | *plce1* | phospholipase C, epsilon 1 ↑ | involved in multiple signaling pathways that can affect cell survival, cell growth, actin organization and T-cell activation |
|  | *ralgds* | ral guanine nucleotide dissociation stimulator ↑ | as a guanine nucleotide exchange factor activating either RalA or RalB GTPases and plays an important role in intracellular transport |
|  | *csf1ra* | colony stimulating factor 1 receptor, a ↑ | plays an important role in innate immunity and in inflammatory processes |
|  | *pla2g10* | phospholipase A_2_ group 10 ↑ | may be involved in maturation and activation of innate immune cells |
|  | *pla2g4c* | cytosolic phospholipase A_2_ gamma-like ↑ | regulate endoplasmic reticulum homeostasis and lipid droplet formation |
|  | *angpt1* | angiopoietin-1-like ↓ | mediate reciprocal interactions |
| Arachidonic acid metabolism | *pla2g4c* | cytosolic phospholipase A_2_ gamma-like ↑ | involved in endoplasmic reticulum membrane homeostasis and lipid droplet biogenesis |
|  | *pla2g10* | phospholipase A_2_ group 10 ↑ | may be involved in maturation and activation of innate immune cells |
|  | *cyp2j4* | cytochrome P450 2J4-like ↑ | catalyzes the hydroxylation of carbon-hydrogen bonds |
|  | *ggt1a* | gamma-glutamyltransferase 1a ↑ | involved in arachidonic acid metabolism |
|  | *—* | hydroperoxide isomerase ALOXE3-like ↑ | oxygenates polyunsaturated fatty acids |
| Ribosome biogenesis in eukaryotes | *gar1* | GAR1 homolog, ribonucleoprotein ↑ | a member of the H/ACA snoRNPs (small nucleolar ribonucleoproteins) family that is responsible for 18 S rRNA production and rRNA pseudouridylation |
|  | *snu13b* | SNU13 homolog, small nuclear ribonucleoprotein b ↑ | a component of the spliceosome and rRNA processing machinery |
|  | *nop58* | NOP58 ribonucleoprotein homolog ↑ | is crucial for rRNA processing and assembly |
